# Semaglutide Protects Retinal Ganglion Cells Against Rotenone-Induced Degeneration via Improved Glucose Metabolism

**DOI:** 10.1167/iovs.67.2.25

**Published:** 2026-02-10

**Authors:** Zaynab A. Mouhammad, James R. Tribble, Alan Nicol, Evgenia Andreopoulou, Mariana Y. García-Bermúdez, Blanca I. Aldana, Rupali Vohra, Miriam Kolko, Pete A. Williams

**Affiliations:** 1 Department of Drug Design and Pharmacology, Faculty of Health and Medical Sciences, University of Copenhagen, Copenhagen, Denmark; 2 Department of Clinical Neuroscience, Division of Eye and Vision, St. Erik Eye Hospital, Karolinska Institutet, Stockholm, Sweden; 3 Department of Veterinary and Animal Sciences, Faculty of Health and Medical Sciences, University of Copenhagen, Copenhagen, Denmark; 4 Department of Ophthalmology, Copenhagen University Hospital, Rigshospitalet, Glostrup, Denmark; 5 Centre for Eye Research Australia, Royal Victorian Eye and Ear Hospital, Melbourne, Australia

**Keywords:** GLP1, semaglutide, glaucoma, mitochondria, rotenone

## Abstract

**Purpose:**

Glaucoma is a multifactorial disease, where metabolic and mitochondrial dysfunction may play a major role in the progressive loss of retinal ganglion cells that characterize the disease. Currently, treatment strategies consist of IOP-lowering approaches with no available neuroprotective agent. In epidemiological studies and models of glaucoma, GLP-1 receptor agonists (GLP-1RAs) reduce the risk of glaucoma and provide protection against the loss of retinal ganglion cells.

**Methods:**

In this study, we explored the potential of semaglutide (SEM), a known GLP-1RA, to protect retinal ganglion cells from rotenone-induced metabolic dysfunction. We pretreated C57BL/6 mice subcutaneously with either SEM (5 mg/kg) or saline solution for one week. After one week, the mice received intravitreal injections of rotenone (10 mM) or dimethylsulfoxide (1%) and were euthanized 24 hours later.

**Results:**

We demonstrated that rotenone caused a significant loss of retinal ganglion cells, which was prevented by SEM pretreatment. Metabolic analyses revealed that SEM enhanced glucose metabolism, which suggested the enhancement of glucose homeostasis/alternative pathways possibly supporting metabolic flexibility of retinal ganglion cells.

**Conclusions:**

SEM may help preserve retinal ganglion cells under conditions of mitochondrial Complex I inhibition, suggesting a potential therapeutic role in glaucoma management; however, further studies are required to confirm metabolic changes observed in this study.

Glaucoma is a chronic optic neuropathy and the most common cause for irreversible blindness, affecting at least 80 million people worldwide.^1^^,^^2^ It is an etiologically complex condition in which retinal ganglion cells (RGCs) experience progressive dysfunction and compartmentalized degeneration with or without elevated IOP.^3^ Although an elevated IOP remains the sole modifiable risk factor, many patients experience severe disease progression despite medical and surgical attempts to lower the IOP^4^^,^^5^; up to 38.1% of patients progress to blindness in at least one eye, and 13.5% are blind in both eyes, 20 years from initial diagnosis,^6^ whereas more than 50% of patients with IOPs within a normal range (normal tension glaucoma) are expected to lose vision despite treatment.^7^^–^^9^ This highlights the critical need to identify neuroprotective approaches that target other potentially modifiable risk factors,^7^ such as metabolic disturbances and mitochondrial dysfunction.^10^^–^^12^ RGCs are particularly sensitive to mitochondrial alterations/metabolic fluctuations,^11^^–^^21^ and increasing evidence from patients and models of glaucoma suggest that disrupted mitochondrial bioenergetics are linked to glaucoma onset and progression.^19^^–^^32^

In this context, glucagon-like-peptide-1 receptor agonists (GLP-1RAs), primarily known for their antidiabetic and weight loss effects, have emerged as promising neuroprotective agents,^33^^,^^34^ showing protective effects on mitochondrial function^35^^,^^36^ and neuronal metabolism.^37^^–^^39^ In models of retinal neurodegeneration, GLP-1RAs normalized mitochondrial membrane potentials, impeded mitophagy, retinal cell loss and protected mitochondria from hyperglycemic and oxidative damage.^35^^,^^40^^,^^41^ GLP-1RAs also protected RGCs from ocular hypertension induced degeneration.^42^^,^^43^ Additionally, according to a Danish,^44^ Taiwanese,^45^ and U.S.^46^ registry-based study, exposure to GLP-1RAs is associated with a reduced risk of glaucoma.

RGCs mainly produce ATP through oxidative phosphorylation^47^ and on low glucose availability, Müller glia (and astrocytes) shuttle alanine and lactate mainly produced by aerobic glycolysis^16^^,^^48^^–^^50^ to support oxidative metabolism of neurons and avoid energetic distress.^51^^–^^53^ In this regard, GLP-1RAs have enhanced oxidative phosphorylation in certain brain regions^37^^,^^54^ and promoted the supportive ability of astrocytes in models of Alzheimer's disease^36^^,^^37^^,^^39^^,^^55^ by enhancing aerobic glycolytic flux.^37^^,^^39^

Given the emerging neuroprotective potential of GLP-1RAs, we aimed to evaluate whether semaglutide (SEM) could protect RGCs against mitochondrial dysfunction-induced degeneration. Specifically, we investigated whether SEM mitigates RGC loss and alters retinal metabolism after intravitreal injection of rotenone (ROT), a mitochondrial Complex I inhibitor. ROT was selected because mitochondrial Complex I-driven ATP production is reduced in patients with glaucoma,^56^ and downregulation of the Complex I NDUFB8 subunit has been observed in GLP-1 receptor knock-out mice,^57^ further linking GLP-1 signaling to mitochondrial health. We focused on SEM not only because of its established efficacy in glycemic control but also because of its dual availability as oral and injectable formulations,^33^ which could facilitate clinical translation if neuroprotective effects are confirmed. In this study, we assess whether semaglutide can protect RGCs against ROT-induced injury and alters retinal metabolism, thereby exploring its potential as a therapeutic strategy in glaucoma and retinal diseases characterized by mitochondrial dysfunction.

## Materials and Methods

### Animal Strain and Husbandry

C57/BL6J female/male mice (aged 8–10 weeks, weighing 20–30*g*) were sourced from SCANBUR, reared and housed at Karolinska Insitutet (Sweden) in a 12-hour light/12-hour dark cycle with food and water available as desired. All breeding and experimental procedures were performed in accordance with the ARVO Statement for the Use of Animals in Ophthalmic and Vision Research and the Danish Act on Animal Experiments (LBKNo. 474 of 15/05/2014). Stockholm's Committee for Ethical Animal Research (10389-2018) approved individual protocols used for the in vivo experiments of this study.

### Animal Model of Metabolic Dysfunction

**Figure 1.**
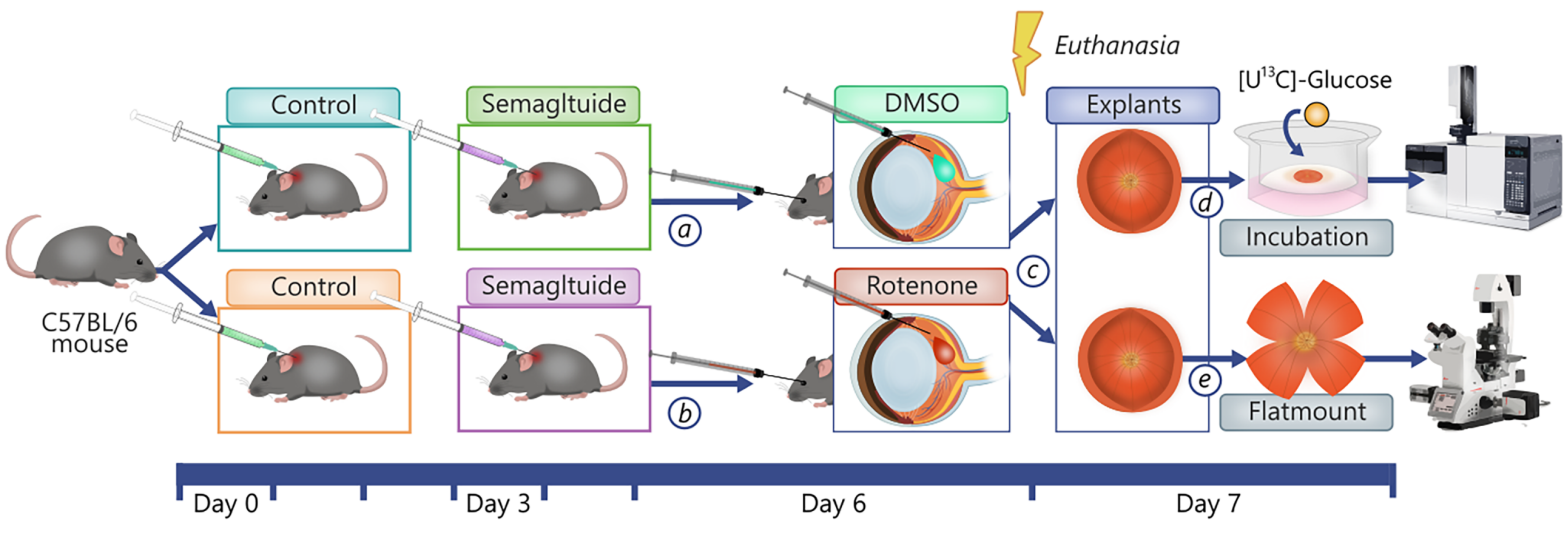
**Experimental timeline and treatment protocol.** C57/BL6J mice were divided into four groups receiving subcutaneous injections of either saline solution (control) or semaglutide on day 0, day 3, and day 6. On day 6, mice also received intravitreal injections of either **(a)** 1% DMSO or **(b)** 10 mM rotenone. **(c)** Mice were euthanized 24 hours after treatment, and eyes were enucleated and isolated as retinal explants or immediately fixed. **(d)** Retinal explants were incubated with labeled glucose and later analyzed by GC-MS. **(e)** Retinal flatmounts were isolated from the fixed eyes, stained with immunofluorescence and imaged.

To induce mitochondrial dysfunction, we used intravitreal injection of ROT as previously described^12^^,^^58^^,^^59^ (Fig. 1). Using a NanoFil 10 µL glass syringe with a 33G needle, we performed bilateral intravitreal injections after anesthetization using an intraperitoneal injection of ketamine (37.5 mg/kg) and midazolam (Dormitol, 1.25 mg/kg). Mice underwent one week of pretreatment with either SEM (5 mg/kg BW, purchased from Nordic Biosite [Täby, Sweden] 98% purity, CAS no 910463-68-2; Adipogen Life Sciences, San Diego, CA, USA) or an equal volume of Hank's balanced salt solution (HBSS; Sigma-Aldrich Corp., St. Louis, MO, USA), injected on day 0, day 3, and day 6 of the study. The dose of SEM was based on the dose of a previous study assessing neuroprotective effects of the GLP-1RA, NLY01, in mice,^43^ and the solution was prepared as a 0.25 mM stock by dissolving 10 mg crystallized SEM in 10 mL HBSS. After one week, mice were anesthetized using an intraperitoneal injection of ketamine (37.5 mg/kg) and midazolam (Dormitol, 1.25 mg/kg) and bilateral intravitreal injections were performed using a NanoFil 10 µL glass syringe with a 33G needle. Mice were bilaterally injected with either 1.5 µL of 10 mM ROT (MP Biochemicals, Fisher Scientific, Waltham, MA, USA) in dimethylsulfoxide (DMSO; PanReac AppliChem, Castellar del Vallès, Spain) or 1.5 µL of DMSO only (control). Twenty-four hours after intravitreal injections, mice were euthanized by cervical dislocation, and retinal tissues were collected for analysis. Thus the ROT-unexposed groups consisted of mice receiving an intravitreal injection of DMSO, treated with either saline solution (DMSO HBSS) or SEM (DMSO SEM). The ROT-exposed groups consisted of mice receiving an intravitreal injection of ROT, treated with either saline solution (ROT HBSS) or SEM (ROT SEM).

### Immunofluorescent Labeling and Imaging of Retinal Flatmounts

Flatmounted retinas were subjected to immunofluorescent labeling. Primary antibody used was anti-RBPMS (anti-RNA-binding protein with multiple splicing; an RGC-specific marker in the retina used only for flatmounts; NBP2-20,112, Rabbit; Novus Biologicals, Littleton, CO, USA), prepared at 2.2 µg/mL. Secondary antibody used was AF568 (A11011, goat anti-rabbit; Invitrogen, Carlsbad, CA, USA), prepared at 4 µg/mL. Both primary and secondary antibodies were diluted in 1 × PBS. For mounting, Fluoromount-G was used, purchased from Invitrogen. Tissues were isolated in 1 × PBS from eyes fixed immediately after enucleation in 3.7% PFA followed by 0.37% PFA. Glass slides were marked with a hydrophobic barrier pen (VWR). Retinal flatmounts were permeabilized in 0.5% Triton X-100 in 1 × PBS for one hour, blocked in 5% BSA in 1 × PBS for one hour, and primary antibody (RBPMS; 1:250) applied overnight at 4°C (at least 20 hours in total). After five washes (five minutes each) with 1 × PBS, a secondary antibody was applied (1:500 in 1 × PBS, RBPMS was matched with Alexa Fluor anti-rabbit 568) for four hours at room temperature. After five washes (five minutes each) in 1 × PBS, excess PBS was removed, and glass coverslips were mounted using Fluoromount-G and sealed with nail-varnish. Images were captured using an epifluorescence microscope (Leica DMi8; Leica, Wetzlar, Germany) at 40× magnification (0.25 µm/pixel), 0–1000 µm from the optic nerve head. Image acquisition was performed at radial positions of 0, 2, 3, 6, 8, and 9 o'clock relative to the optic nerve head. Each image was cropped to a 150 × 150 µm area for cell counting. RBPMS+ cells were counted using the Cell Counter plugin in FIJI (ImageJ)^60^ and normalized to 0.01 mm^2^. The diameters of RGCs were measured using the straight-line tool on FIJI, with measurements taken across the widest part of each cell. For each retina, six images were analyzed, and 17 RGCs were measured per image, yielding approximately 100 cells measured per retina.

#### Functional Metabolic Mapping of Glucose Metabolism in Retinal Explants

Immediately after enucleation, retinal explants were isolated and pre-incubated on permeable membrane cell culture inserts (MilliCell Standing Inserts, 6-well format, 0.4 µm pore size, PICM03050; Merck, Darmstadt, Germany) in a humidified incubator with 5% CO_2_/ 95% O_2_ at 37°C for 30 minutes with preheated glucose-free supplemented media containing Neurobasal A supplemented with: 2% B-27 supplement (50×; Fisher Scientific), 1% N-2 supplement (100×; Fisher Scientific), 1% penicillin–streptomycin (10,000 U/mL; Gibco, Thermo Fisher Scientific), and 0.1% amphotericin B (Gibco, Thermo Fisher Scientific). Retinal explants were then washed once with 1 × PBS and incubated for 30 minutes with supplemented media containing labeled glucose, 6 mM [U-^13^C]-glucose isotope (cat. no. CLM-1396-10, L-isomer of 98% chemical purity; Cambridge Isotopes Laboratories Inc., Tewksbury, MA, USA), 2% B-27 supplement (50×; Fisher Scientific), 1% N-2 supplement (100×; Fisher Scientific), 1% penicillin–streptomycin (10,000 U/mL; Gibco, Thermo Fisher Scientific), and 0.1% amphotericin B (250 µg; Gibco, Thermo Fisher Scientific) amphotericin B/mL. After 30 minutes, retinal metabolism was halted by washing the retinal explants with ice-cold 1 × PBS and terminated with ice cold 96% ethanol. Retinal samples were kept on ice, sonicated, spun in a centrifuge (20,000*g*, 20 minutes at 4°C), distributed into new labeled tubes and stored at −80°C for at least 24 hours before lyophilization. Lyophilized supernatants were stored at −20°C until further spectrometric analysis. Spectrometric analyses were performed using a gas chromatograph (7820A; Agilent Technologies, Winooski, VT, USA) equipped with a J&W GC column (HP-5MS, part no. 19091S-433; Agilent Technologies) and coupled to a mass spectrometer (5977E; Agilent Technologies) to determine the ^13^C-enrichment in several cellular metabolites. Lyophilized supernatants were reconstituted in distilled water, acidified with HCl to pH 1-2 and evaporated under nitrogen flow. Using 96% ethanol and benzene, analytes were extracted into an organic phase and subsequently derivatized using 14% dimethylformamide and 86% N-(t-butyldimethylsilyl)-N-methyltrifluoroacetamide. A standard containing metabolites of interest was also prepared similarly to extracts from the retinal explants. The isotopic enrichment of the metabolites of interest was corrected for the natural abundance of ^13^C using the unlabeled standards and calculated according to Biemann et al.^61^ Twelve metabolites that could be verified with standards were detected. Data were analyzed using the Agilent MassHunter Quantitative Data Analysis software. Data are presented as labeling (%) of M+X, where M is the mass of the unlabeled molecule and X is the number of ^13^C-labeled carbon atoms in each metabolite.

### Glutathione Quantification

Mouse retinas were collected, snap-frozen immediately after dissection, and stored at −80°C until processing. For glutathione analysis, each frozen retina was placed on ice and transferred to a pre-chilled 1.5 mL tube. A total of 100 µL HBSS was added, and tissues were homogenized by brief sonication (three- to five-second pulses) followed by vortex mixing. All steps were performed on ice to minimize artifactual oxidation. After homogenization, 5 µL of lysate was removed for the glutathione assay. Because preliminary tests revealed that the manufacturer-recommended 25 µL sample input produced luminescence values outside the linear range for retinal tissue, samples were diluted to a final assay volume of 25 µL by adding 20 µL HBSS. Total glutathione (GSH + GSSG), oxidized glutathione (GSSG), reduced glutathione (GSH), and the GSH/GSSG ratio were quantified using the GSH/GSSG-Glo Assay (Promega Corporation, Madison, WI, USA; Cat. V6611/V6612). The assay was performed according to the manufacturer's instructions. For each sample, 25 µL of diluted lysate was dispensed into white opaque 96-well plates. To measure total glutathione, wells received 25 µL total glutathione lysis reagent; to measure oxidized glutathione, separate wells received 25 µL Oxidized Glutathione Lysis Reagent, which includes N-ethylmaleimide to block reduced GSH. Plates were mixed for five minutes at room temperature. Subsequently, 50 µL Luciferin Generation Reagent was added to all wells, followed by a 30-minute incubation at room temperature. Luminescence was initiated by adding 100 µL Luciferin Detection Reagent and stabilizing for 15 minutes before measurement on a microplate luminometer. All reagents were prepared immediately before use, as recommended. The assay produces luminescence proportional to glutathione content. Total GSH (µM), oxidized GSH (µM), and reduced GSH (µM) were obtained directly from assay output using standard curves. The GSH/GSSG ratio was calculated according to the equations provided in the manufacturer's instructions.

#### Analysis and Statistics

All statistical analyses were performed in GraphPad Prism 10.3.1 (GraphPad, San Diego, CA, USA). Normality of data was tested with the Shapiro-Wilk test. When comparing two conditions, normally distributed data was analyzed by one-way ANOVA followed by post-hoc Tukey's multiple comparison test to evaluate statistical differences between experimental groups. Homogeneity of variance was evaluated using Bartlett's test. If Bartlett's test was significant (*P* < 0.05), the assumption of equal variances was considered violated; therefore, Welch's correction was applied, and Welch's ANOVA (or Welch's *t*-test for two groups) was performed. Outliers were detected using Grubb's outlier test and subsequently removed from further analyses. In all analyses, *P* < 0.05 was considered statistically significant. For the box plots, the center hinge represents the mean, and upper and lower hinges represent the first and third quartiles; whiskers represent 1.5 times the interquartile range. Inkscape version 1.3.2 was used to illustrate figures presented in this article.

## Results

### Semaglutide Impedes Rotenone-Induced RGC Loss

**Figure 2.**
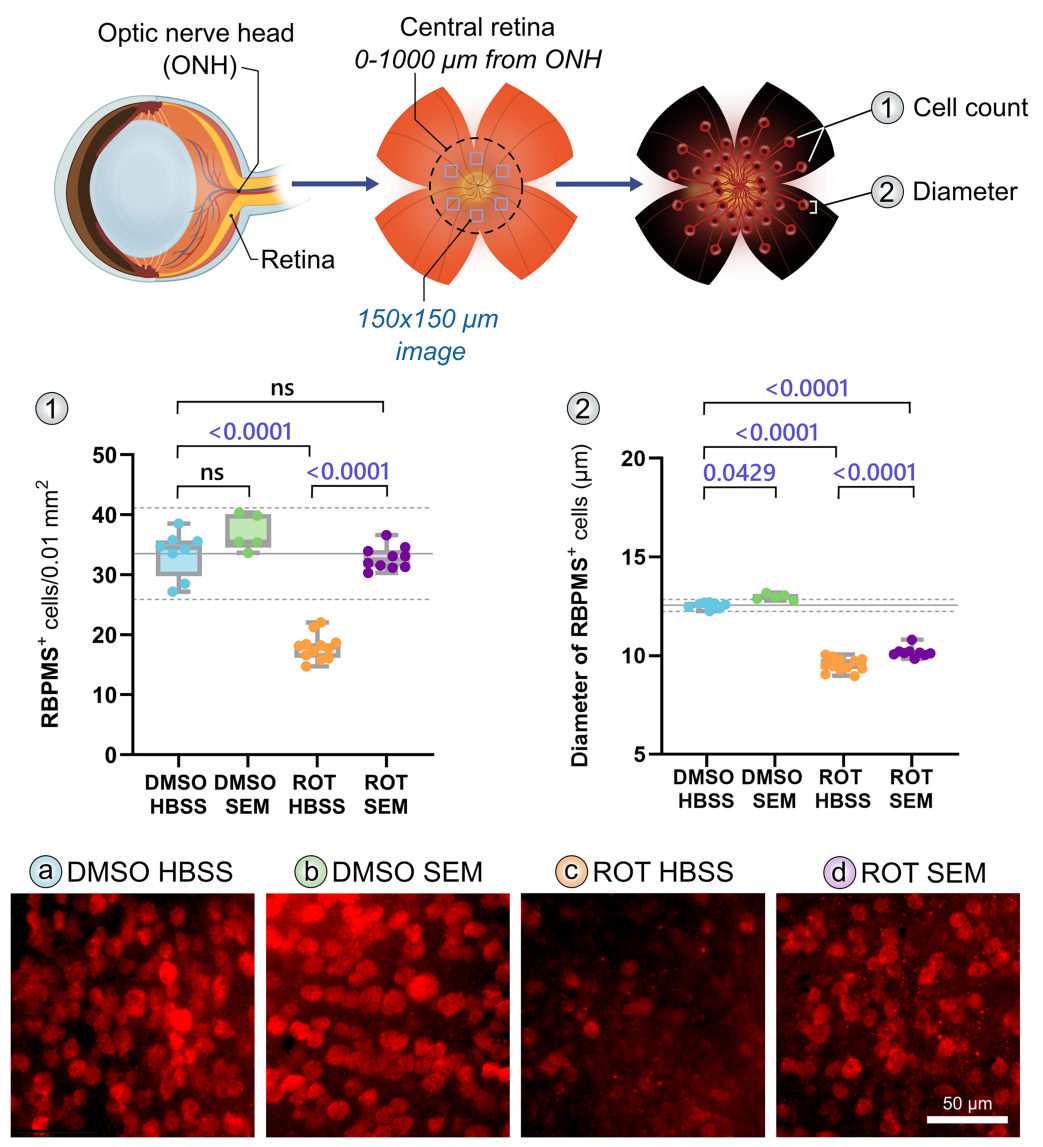
**SEM protects from rotenone induced RGC degeneration.** C57BL6/J wild-type (WT) were treated with either saline (HBSS) or semaglutide (SEM). After one week, mice were subjected to intravitreal injection of 1% DMSO or 10 mM rotenone. 24 hours later, retinas were collected after euthanasia and prepared as flatmounts. Retinal ganglion cells (RGCs) were immunolabeled with RBPMS and quantified. Central retinal regions located 0–1000 µm from the optic nerve head (ONH) were imaged using 150 × 150 µm fields. Data shown represent quantification of (1) RBPMS^+^ cell density and (2) RBPMS^+^ cell soma diameter. The results suggest that SEM may significantly prevent rotenone-induced RGC loss and soma shrinkage. Representative retinal images are shown for (**a**) DMSO HBSS, (**b**) DMSO SEM, (**c**) ROT HBSS, and (**d**) ROT SEM. *Scale bar*: 50 µm. Statistical analysis was performed using one-way ANOVA followed by post hoc Tukey's multiple comparison test. Sample sizes: DMSO: *n* = 8, SEM-DMSO: *n* = 5, ROT: *n* = 12, SEM-ROT: *n* = 10 retinas. Included comparisons are DMSO HBSS vs. DMSO SEM, ROT HBSS and ROT SEM, and ROT HBSS vs. ROT SEM. HBSS, Hanks' balanced salt solution; ONH, optic nerve head; SEM, semaglutide; ROT, rotenone.

To determine if SEM provides neuroprotection to RGCs after metabolic disruption, we induced degeneration through an intravitreal injection of ROT and assessed RGC viability (Fig. 2). ROT induced a significant RGC loss (ROT HBSS) compared to the control (DMSO HBSS; *P* < 0.0001, analyzed by one-way ANOVA plus post-hoc Tukey's test). Compared to untreated mice (ROT HBSS), SEM pretreatment (ROT SEM) before any insult caused a significantly superior RGC viability (*P* < 0.0001). Because we used DMSO as our solvent for our ROT solution, the control mice received intravitreal injections of DMSO and were similarly treated with either saline solution (DMSO HBSS) or SEM (DMSO SEM). In these groups, one-week SEM pretreatment, before any insult did not induce any difference in RGC viability (*P* = 0.1287).

### Glucose Metabolism in Acutely Isolated Retinas

**Figure 3.**
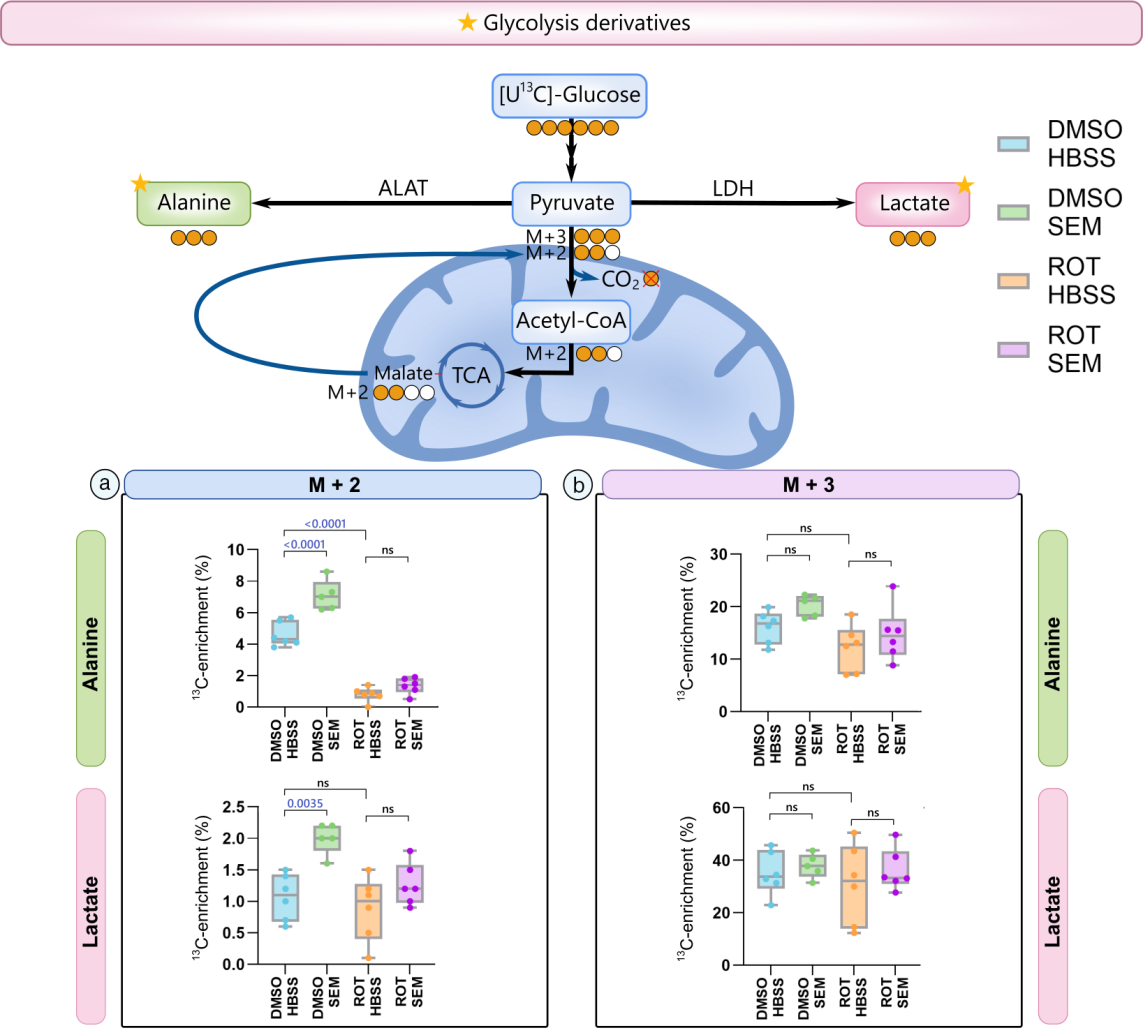
**SEM improves metabolism of lactate and alanine M+2 in acutely isolated retinas (*n* = 5–6).** Intracellular ^13^C enrichment in alanine and lactate was assessed in retinal explants following metabolism of 6 mM [sU-^13^C]glucose. C57BL6/J wild-type (WT) mice were treated subcutaneously with either saline (HBSS) or semaglutide (SEM) and subjected to intravitreal injections of either 1% DMSO or 10 mM rotenone (ROT) 24 hours before euthanasia. Retinal metabolism was analyzed using [U-^13^C]glucose and gas chromatography-mass spectrometry (GC-MS). Metabolism of [U-^13^C]glucose via glycolysis generates the glycolytic end product pyruvate M+3. Through the enzymes alanine aminotransferase (ALAT) and lactate dehydrogenase (LDH), pyruvate M+3 can be converted to alanine M+3 and lactate M+3, and ^13^C enrichment in these metabolites therefore reflects glycolytic activity. Alanine and lactate M+2 may arise from pyruvate M+2, which can be derived from malate M+2 via the tricarboxylic acid (TCA) cycle. Data shown represent quantification of ^13^C enrichment for (**a**) M+2 isotopologues and (**b**) M+3 isotopologues of alanine (top panels) and lactate (bottom panels). SEM treatment significantly increased M+2 ^13^C-enrichment in alanine and lactate in retinas exposed to DMSO compared with saline treated mice. In the rotenone exposed retinas from SEM-treated mice, ^13^C enrichment in alanine M+2 was also higher than in saline treated mice, but not as a variable. Data represent the means, *n* = 5–6 retinas per group. Statistical analysis was performed using one-way ANOVA followed by Tukey's multiple-comparison test. ALAT, alanine aminotransferase; HBSS, Hanks' balanced salt solution; LDH, lactate dehydrogenase; SEM, semaglutide; TCA, tricarboxylic acid cycle; ROT, rotenone.

Given that GLP-1RAs alter cellular metabolism, we next assessed whether the observed changes in RGC survival after treatment with SEM were directly linked to favorable alterations in retinal metabolism. To examine this, we repeated our previous paradigm (where mice were pretreated for a week with SEM or HBSS before intravitreal injection of ROT or DMSO), but retinas were maintained in ex vivo culture after enucleation at the 24-hour post-intravitreal injection time point. Retinal explants were incubated with 6 mM [U-^13^C]glucose on inserts to prevent hypoxia, which allowed assessment of metabolic flux under near normoxic conditions. Following uptake, [U-^13^C]glucose is metabolized through glycolysis to produce fully labeled pyruvate (M+3), which can be transaminated to alanine M+3 by alanine aminotransferase or reduced to lactate M+3 by lactate dehydrogenase (Fig. 3). In mitochondria, pyruvate M+3 can be decarboxylated to acetylCoA M+2 by pyruvate dehydrogenase, and subsequently integrated into the TCA cycle intermediates, including malate M+2. Through mitochondrial oxidative decarboxylation, malate M+2 can also be converted to pyruvate M+2. This ultimately gives rise to alanine and lactate M+2.

No significant differences were observed in the ^13^C-enrichment in lactate and alanine from mice exposed to ROT and DMSO in M+3 (Fig. 3B), and SEM treatment similarly did not cause a significant change in ^13^C-enrichment. In M+3, ROT exposure did not cause a significant reduction in ^13^C labeling compared to DMSO in both SEM and saline treated mice. The relative ^13^C-enrichment of both alanine M+2 and lactate M+2 (Fig. 3A) was found to be higher in retinal extracts of SEM treated mice exposed to DMSO, when compared to saline solution–treated mice (*P* < 0.0001, and *P* = 0.0035). Furthermore, ROT exposure did not lead to a significant reduction in ^13^C enrichment of alanine nor lactate, except for alanine M+2 (*P* < 0.0001), compared to saline solution–treated mice exposed to DMSO.

**Figure 4.**
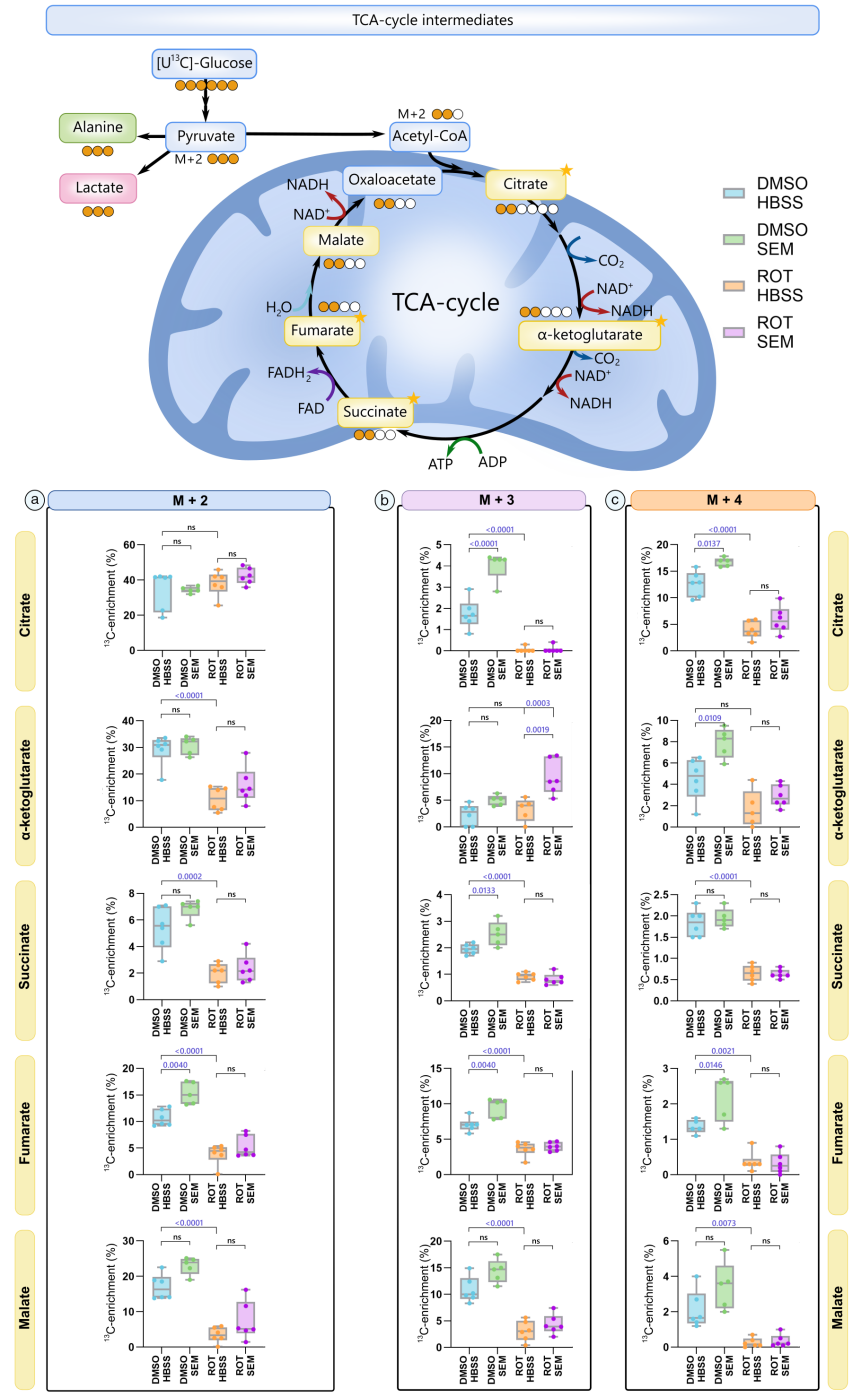
**SEM increases flux of carbon through the TCA cycle in acutely isolated retinas (*n* = 5–6).** Intracellular ^13^C enrichments of TCA cycle intermediates and associated amino acids. M+2 labeling reflects the first-turn metabolism of [U-^13^C]glucose, and M+3/M+4 labeling the second-turn metabolism. Data shown represent quantification of ^13^C enrichment for (**a**) M+2, (**b**) M+3, and (**c**) M+4 isotopologues of TCA cycle intermediates. In mice exposed to intravitreal injections of DMSO, semaglutide (SEM) treatment significantly increased ^13^C enrichment in fumarate M+2/M+3/M+4, citrate M+3/M+4, α-ketoglutarate M+4 and succinate M+3. In contrast, rotenone exposure significantly reduced ^13^C enrichment across all measured TCA cycle intermediates, except for α-ketoglutarate, compared to DMSO-exposed saline treated mice (DMSO HBSS). Although SEM treatment appeared to increase ^13^C labeling in several metabolites in rotenone-exposed retinas (ROT HBSS vs. ROT SEM), the only statistically significant change was observed in α-ketoglutarate M+3. Data represent the means, *n* = 5–6, one-way ANOVA followed by Tukey's multiple comparison test. HBSS, Hanks' balanced salt solution; SEM, semaglutide; ROT, rotenone.

**Figure 5.**
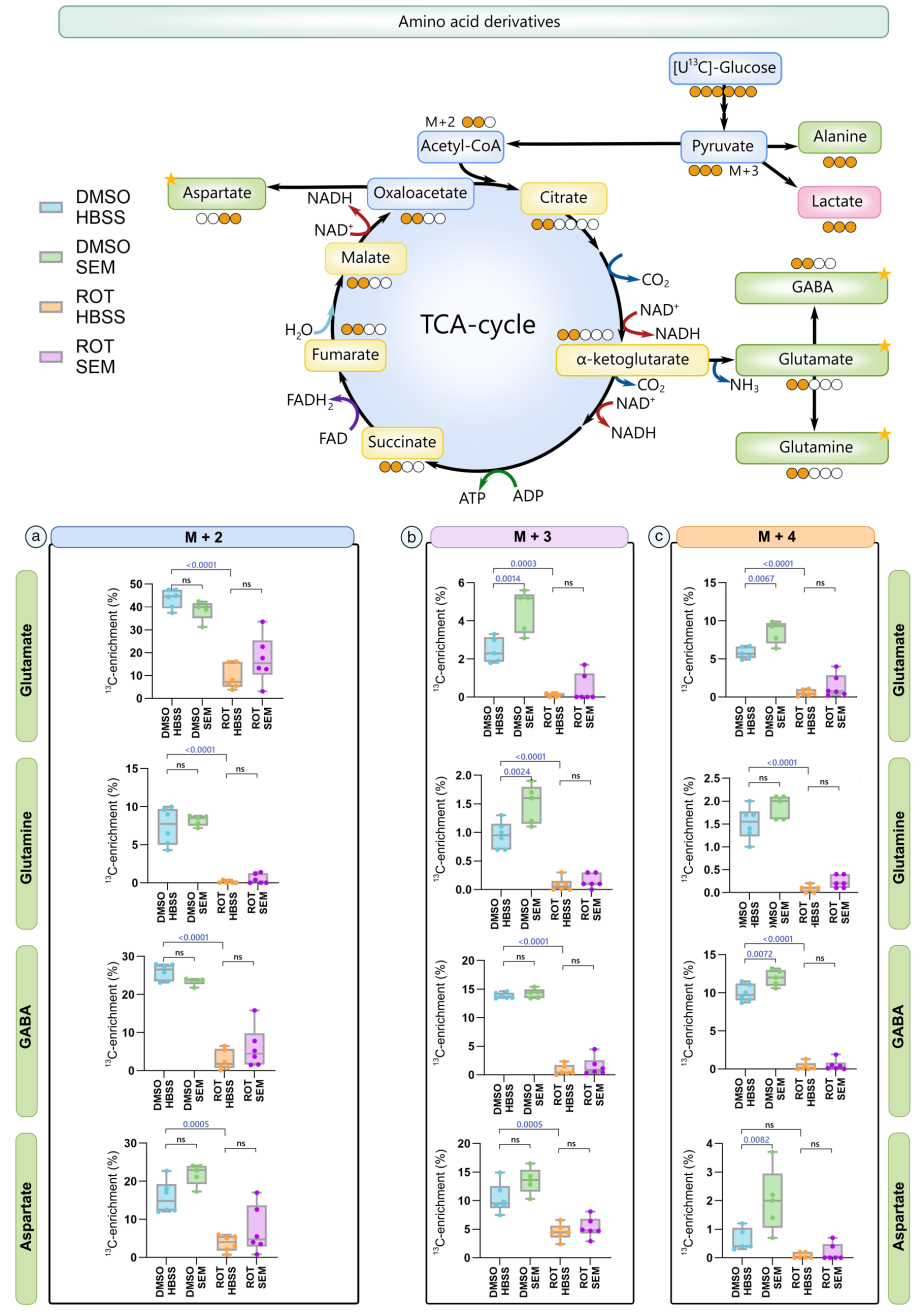
**SEM alters amino acid isoptologues in acutely isolated retinas (*n* = 5–6).** Intracellular ^13^C enrichments in TCA cycle-associated amino acids. M+2 labeling reflects a first turn of the TCA cycle and derived labeling, M+3/M+4 labeling represents labeling in a second turn. Data represent quantification of ^13^C enrichment for (**a**) M+2, (**b**) M+3, and (**c**) M+4 isotopologues of TCA cycle-associated amino acids. In retinas exposed only to DMSO, semaglutide (SEM) treatment significantly increased ^13^C enrichment of glutamate M+3, glutamine M+3, GABA M+4, and aspartate M+4 compared to mice treated with saline (HBSS). SEM treatment did not significantly alter ^13^C enrichment of any TCA cycle associated amino acid compared to untreated mice, when retinas were exposed to rotenone (ROT). However, SEM treatment of ROT exposed retinas seemed to induce a higher ^13^C enrichment in several amino acids (e.g., glutamate M+2, GABA M+2, Aspartate M+2), but not as a variable. GABA, γ-aminobutyric acid; HBSS, Hanks' balanced salt solution; SEM, semaglutide; ROT, rotenone; TCA, tricarboxylic acid cycle.

When pyruvate M+3 is converted to acetyl CoA M+2 by pyruvate dehydrogenase in the mitochondria, it can subsequently be integrated into the TCA cycle intermediates (Fig. 4) and its associated amino acids (Fig. 5) as M+2 in the first turn and M+3/M+4 in the second turn of the TCA cycle. However, M+3 and M+4 isotopologues may also arise through mixing with unlabeled carbon atoms from preexisting metabolite pools, anaplerotic inputs (e.g., pyruvate carboxylase), or turnover of larger metabolic reservoirs. Thus higher-order isotopologues reflect both continued ^13^C incorporation and dilution from unlabeled substrates

Overall, SEM-treatment increased ^13^C enrichment from metabolism of [U-^13^C]glucose when compared to mice treated with saline. However, after the first turn of the TCA cycle, treatment with SEM did not alter M+2 labeling of any TCA-cycle intermediates nor associated amino acids, except for fumarate M+2 (*P* = 0.0040) in retinas exposed only to DMSO. Except for citrate M+2 (*P* = 0.8484), ROT caused a significant decrease in M+2 ^13^C-enrichment in all measured TCA cycle intermediates and associated amino acids regardless of whether or not the mice were treated with SEM.

After second-turn metabolism of [U-^13^C]glucose (Figs. 4B, 5B), SEM caused a significantly higher ^13^C enrichment in citrate (*P* < 0.0001), succinate (*P* < 0.0133), fumarate (*P* < 0.004), glutamate (*P* < 0.0014), and glutamine (*P* < 0.0024) M+3 in retinas exposed to DMSO compared to retinas from mice treated with saline solution. Similar to M+2, ROT exposure decreased ^13^C labeling in all measured TCA cycle intermediates and associated amino acids in M+3, except for α-ketoglutarate (α-KG) M+3. For α-KG M+3, SEM treatment led to an increased ^13^C labeling in mice exposed to ROT compared to saline solution–treated mice (*P* = 0.0019). In M+4 (Figs. 4C, 5C), treatment with SEM caused a significantly higher ^13^C enrichment in citrate (*P* = 0.0124), fumarate (*P* = 0.0146), glutamate (*P* = 0.0067), GABA (*P* = 0.0072), and aspartate M+4 (*P* = 0.0082).

### Rotenone-Induced Oxidative Stress Lowers GSH/GSSG Ratio in Mouse Retina

**Figure 6.**
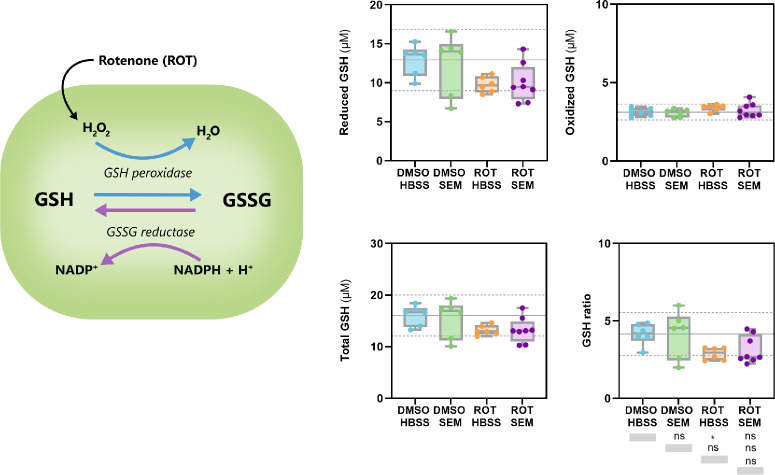
**Rotenone disrupts retinal glutathione redox balance with limited protection by semaglutide (*n* = 5–6).** Reduced, oxidized, and total glutathione concentrations, as well as the resulting GSH/GSSG ratio, were quantified in homogenized mouse retinas to evaluate changes in redox homeostasis following systemic SEM treatment and intravitreal ROT exposure. Rotenone injection markedly decreased reduced GSH in HBSS-treated retinas compared with the DMSO HBSS control group, confirming a depletion of the antioxidant pool. Although SEM-treated retinas exposed to ROT (ROT SEM) showed slightly higher reduced GSH than HBSS+ROT, this effect did not reach statistical significance. In contrast, oxidized GSH levels remained low across all conditions and did not differ significantly between treatment groups. Consistent with these findings, total glutathione content was significantly reduced by ROT, with only modest, non-significant preservation observed in SEM-treated mice. The GSH/GSSG ratio, a sensitive indicator of intracellular redox state, was significantly decreased in HBSS+ROT retinas relative to DMSO HBSS controls, indicating impaired antioxidant capacity. Semaglutide did not significantly restore the redox ratio under ROT challenge, although a mild upward trend was observed. Together, these data demonstrate that ROT induces oxidative stress in the retina by depleting reduced GSH and lowering the GSH/GSSG ratio, whereas SEM does not confers significant protection. Data represent means from *n* = 5–6 retinas per group, analyzed by one-way ANOVA followed by Tukey's post-hoc test.

To determine whether systemic semaglutide modulates retinal redox homeostasis under mitochondrial stress, we quantified reduced glutathione (GSH), oxidized glutathione (GSSG), total glutathione, and the GSH/GSSG ratio in retinas from mice treated with semaglutide or HBSS and challenged intravitreally with either ROT or DMSO. As illustrated in Figure 6, ROT disrupts mitochondrial electron transfer and promotes conversion of GSH to GSSG, thereby lowering the intracellular redox ratio. Across conditions, reduced GSH levels were significantly decreased by ROT, as shown by the marked reduction in the HBSS+ROT group compared with vehicle-treated controls (HBSS+DMSO). SEM-treated retinas exposed to ROT (SEM+ROT) showed partially preserved reduced GSH, although this did not fully reach control levels (Fig. 6). In contrast, oxidized GSH levels remained relatively low and did not differ significantly between treatment groups, indicating that ROT primarily depleted the reduced pool rather than markedly increasing GSSG accumulation (Fig. 6). Consistent with these findings, total glutathione was reduced after ROT injection, with the largest decrease observed in the HBSS+ROT group. Semaglutide again showed a modest but nonsignificant tendency to attenuate ROT-induced depletion (Fig. 6). The GSH/GSSG ratio was significantly lowered by ROT, confirming impaired antioxidant capacity. SEM treatment did not significantly restore the redox ratio under ROT challenge. Collectively, these results demonstrate that ROT induces clear oxidative stress in the retina by reducing the available pool of reduced GSH and lowering the GSH/GSSG ratio, whereas SEM provides no significant protection of retinal glutathione homeostasis under these conditions.

## Discussion

The retina is among the most metabolically active tissues in the body, relying on a constant supply of energy substrates to sustain its high metabolic demands.^62^^–^^65^ In the present study, we assessed the neuroprotective effects of SEM, a GLP-1RA, in a mouse model with metabolic dysfunction. We identified that SEM offers neuroprotection to RGCs possibly by improving the retina's metabolic flexibility. We caused metabolic dysfunction and mitochondrial compromise implicated in a range of retinal diseases, including glaucoma,^66^ by intravitreal injection of ROT, after one-week pretreatment with SEM or saline solution (HBSS). Inhibition of mitochondrial Complex I, as exerted by ROT, is associated with the accumulation of ROS, mitochondrial and endoplasmic reticulum stress and a potent loss of retinal cells and RGCs.^58^^,^^62^^,^^67^ We observed a significant decrease in RGC viability and diameter 24 hours after ROT exposure. However, pretreatment with SEM significantly enhanced the viability and preserved the diameter of ROT-exposed RGCs. These results are consistent with previous findings, in which exenatide, dulaglutide, liraglutide, and other GLP-1RA protected against ROT-induced degeneration of dopaminergic neurons in the striatum^68^^,^^69^ and cerebellar Purkinje cells.^70^ Furthermore, the GLP-1RAs, NLY01, liraglutide, and lixisenatide demonstrated effectiveness against RGC degeneration in mouse models of hypertensive glaucoma^42^^,^^43^ and other models of retinal degeneration.^71^^,^^72^

GLP-1RAs are believed to cause neuroprotection by reducing oxidative stress,^35^^,^^71^^,^^73^ inflammation,^42^^,^^43^^,^^72^^,^^74^ apoptosis,^72^^,^^75^ and improve mitochondrial survival (Refs. 35, 40, 41), regeneration (40), integrity (40) and function (35) in retinal neurons and glial cells.^74^ GLP-1RAs could also contribute to neuroprotection by inducing favorable metabolic adaptations, given that GLP-1 receptor signaling appears to play an important role in brain glucose metabolism.^76^ However, studies on the effects of GLP-1 and its receptor agonists on retinal glucose metabolism remain limited. Using functional metabolic mapping, we were able to identify alterations in the relative levels of intermediate metabolites linked to both glycolysis and the TCA cycle after treatment with SEM and exposure to ROT. In DMSO-exposed retinas, SEM treatment, compared with saline, was associated with increased ^13^C-labeling in glycolytic derivates (alanine M+2 and lactate M+2), TCA cycle intermediates (citrate M+3/M+4, α-ketoglutarate M+4, succinate M+3, fumarate M+2/M+3/M+4) and associated amino acids (glutamate M+3/M+4, glutamine M+3, GABA M+4 and aspartate M+4). These labeling patterns generally suggest increased retinal glucose uptake, but also increased flux of carbon through glycolysis and the TCA cycle, with possible contributions from anaplerotic and cataplerotic processes, on SEM treatment. GLP-1RAs have previously been shown to enhance GLUT-1-mediated glucose transport across the blood-brain barrier^77^^,^^78^ and to increase hexokinase activity,^78^ thereby promoting intracellular trapping of glucose as glucose-6-phosphate. The increased ^13^C enrichment in lactate and alanine in SEM-treated mice suggests increased glycolytic flux, and may also reflect an increased availability of these metabolites to serve as alternative energy substrates through glial (Müller glia and astrocytic) support of retinal neuronal metabolism. This observation contrasts with the only previous study of lixisenatide in the retina, which reported no effect on glycolysis.^79^ However, glycolytic activity in that study was assessed indirectly through methylglyoxal, a glycolytic by-product to assess ROS production, whereas our tracer-based approach directly captured carbon flow. Moreover, findings from other neurodegeneration models show that GLP-1RAs and semaglutide increase glycolytic enzyme expression, enhance aerobic glycolysis, and suppress oxidative phosphorylation in astrocytes through PI3K/Akt and SIRT1/GLUT4 signaling,^37^^,^^39^ supporting a role of GLP-1RAs in enhancing glycolysis. Notably, lactate M+2 enrichment in SEM-treated, DMSO-exposed retinas may also reflect carbon shunting into the oxidative branch of the pentose-phosphate pathway (PPP). This enrichment could arise either from increased glycolytic conversion of glucose to pyruvate or from diversion of glucose-6-phosphate into the PPP, the latter generating NADPH to sustain antioxidant defense under conditions of mitochondrial stress. Because DMSO can impair mitochondrial respiration and increase ROS,^80^ diversion of glucose-6-phosphate into the PPP would provide NADPH for antioxidant defense.^81^^,^^82^ Thus part of the lactate M+2 signal under these conditions may indicate a protective metabolic adaptation supporting redox homeostasis. It is also worth noting that 80–90% of retinal glycolysis originates from photoreceptors.^83^ Although our whole-retina measurements cannot resolve cell specificity, the observed changes are consistent with the possibility of GLP-1RA-mediated effects on photoreceptor metabolism, which to our knowledge remain unexplored.

The increased ^13^C labeling observed in several TCA cycle intermediates upon SEM treatment suggests increased flux of carbon through mitochondrial pathways. These changes may indicate enhanced oxidative metabolism, but may also reflect contributions from anaplerotic inputs (e.g., pyruvate carboxylase activity) and cataplerotic efflux into amino acid synthesis. Similar effects have been reported with liraglutide, which stimulates Complex I activity and multiple TCA cycle enzymes via the SIRT1 pathway.^73^ Given that GLP-1R signaling is known to increase glucose uptake,^33^^,^^37^ the observed enrichment may result from greater substrate availability in addition to changes in mitochondrial enzyme activity. The absence of significant alanine and lactate M+3 enrichment suggests that enhanced labeling of TCA intermediates is not solely driven by increased glycolytic input. Together with preserved RGC viability, these findings are consistent with SEM supporting mitochondrial resilience and metabolic flexibility, although direct measures of mitochondrial function are needed to confirm this.

With respect to amino acid production, increased ^13^C enrichment of aspartate in SEM-treated retinas suggests greater oxaloacetate turnover and enhanced activity of the malate-aspartate shuttle. SEM also increased ^13^C labeling of glutamate and glutamine, indicating altered routing of carbon into the glutamate-glutamine pathway. Such changes are compatible with enhanced neurotransmitter cycling and increased glial uptake of glutamate, processes that in both brain and retina contribute to buffering excitotoxicity and maintaining metabolic stability.^84^^–^^93^ Glial glutamate uptake also supplies substrate for glutamine synthesis and, indirectly, for gluthathione biosynthesis.^92^^,^^93^ Although SEM increased labeling of glutamate and glutamine in our tracer experiments, these metabolic adaptations did not translate into significant changes in reduced GSH, oxidized GSH, or total glutathione, nor did they prevent the ROT-induced reduction in the GSH/GSSG ratio. This contrasts with previous studies reporting that GLP-1RAs protect RGCs and Müller glia from oxidative stress.^40^^,^^94^^,^^95^ Thus, although SEM clearly modulates amino acid and neurotransmitter-linked carbon flux, these shifts appear to support metabolic and neurotransmitter resilience rather than restoring the glutathione redox system under acute mitochondrial stress. Increased GABA labeling in SEM-treated retinas indicates altered flux through the glutamate-glutamine-GABA pathway. Because both GABA and glutamate decline with glaucoma progression,^96^ such changes may enhance retinal resilience. Consistently, topical exendin-4, another GLP-1RA, has been shown to mitigate excitotoxic damage by increasing GABA, promoting RGC survival.^97^

By contrast, ROT markedly suppressed retinal glucose metabolism, as reflected by decreased labeling of most TCA intermediates relative to DMSO controls. Notably, SEM preserved carbon flow into α-KG M+3, which may indicate a metabolic adaptation. This labeling could arise from citrate M+3 formed by condensation of acetyl-CoA M+2 with oxaloacetate M+1, or from glial pyruvate carboxylase activity generating oxaloacetate M+3.^98^ Because α-KG is in rapid exchange with glutamate in the glutamate–glutamine cycle, its increased ^13^C enrichment may also reflect neurotransmitter-linked flux. Under conditions of impaired electron transport, early TCA steps can be repurposed toward neurotransmitter cycling and redox support rather than ATP production.^99^^–^^102^ Although glutamine-derived α-KG can in principle fuel substrate-level phosphorylation at succinyl-CoA synthetase,^103^^,^^104^ we observed no succinate enrichment, supporting a role in anaplerosis and cataplerosis rather than bioenergetics. Such metabolic adaptation, in which α-KG contributes to NADH and NADPH regeneration independently of oxidative phosphorylation,^105^ may help support neuronal survival during mitochondrial stress characteristic of glaucomatous neurodegeneration.^106^^–^^108^ However, despite this preserved α-KG-linked flux, these metabolic shifts did not translate into significant alterations in reduced GSH, oxidized GSH, or total glutathione, nor did SEM prevent the ROT-induced reduction in the GSH/GSSG ratio. Thus, although SEM appears to maintain aspects of mitochondrial and amino acid–linked metabolic resilience under complex I inhibition, these adaptations were not sufficient to normalize retinal glutathione redox balance in our experimental model. Taken together, our findings indicate that SEM promotes glycolytic and TCA-cycle adaptations and supports neurotransmitter-linked carbon flux, thereby fostering a metabolically resilient retinal environment. The associated preservation of RBPMS+ cell counts suggests that these metabolic adaptations contribute to neuroprotection. However, these shifts did not significantly restore glutathione redox homeostasis, emphasizing that SEM's neuroprotective effects in this model likely arise from metabolic rather than glutathione-mediated antioxidant mechanisms. Clinically, semaglutide's availability in both oral (Rybelsus) and injectable (Ozempic, Wegovy) formulations highlights its potential as an adjunct to existing glaucoma therapies. Nonetheless, several limitations must be noted: analyses were performed in whole-retina explants, which mask cell-type-specific contributions; DMSO itself can impair mitochondrial function^80^^,^^109^; and uninjected controls were not included. Future studies should address these gaps by employing purified cultures of RGCs, Müller glia, and photoreceptors, testing lower ROT concentrations and quantifying absolute metabolite levels to better define semaglutide's neuroprotective potential.

## Conclusions

**Figure 7.**
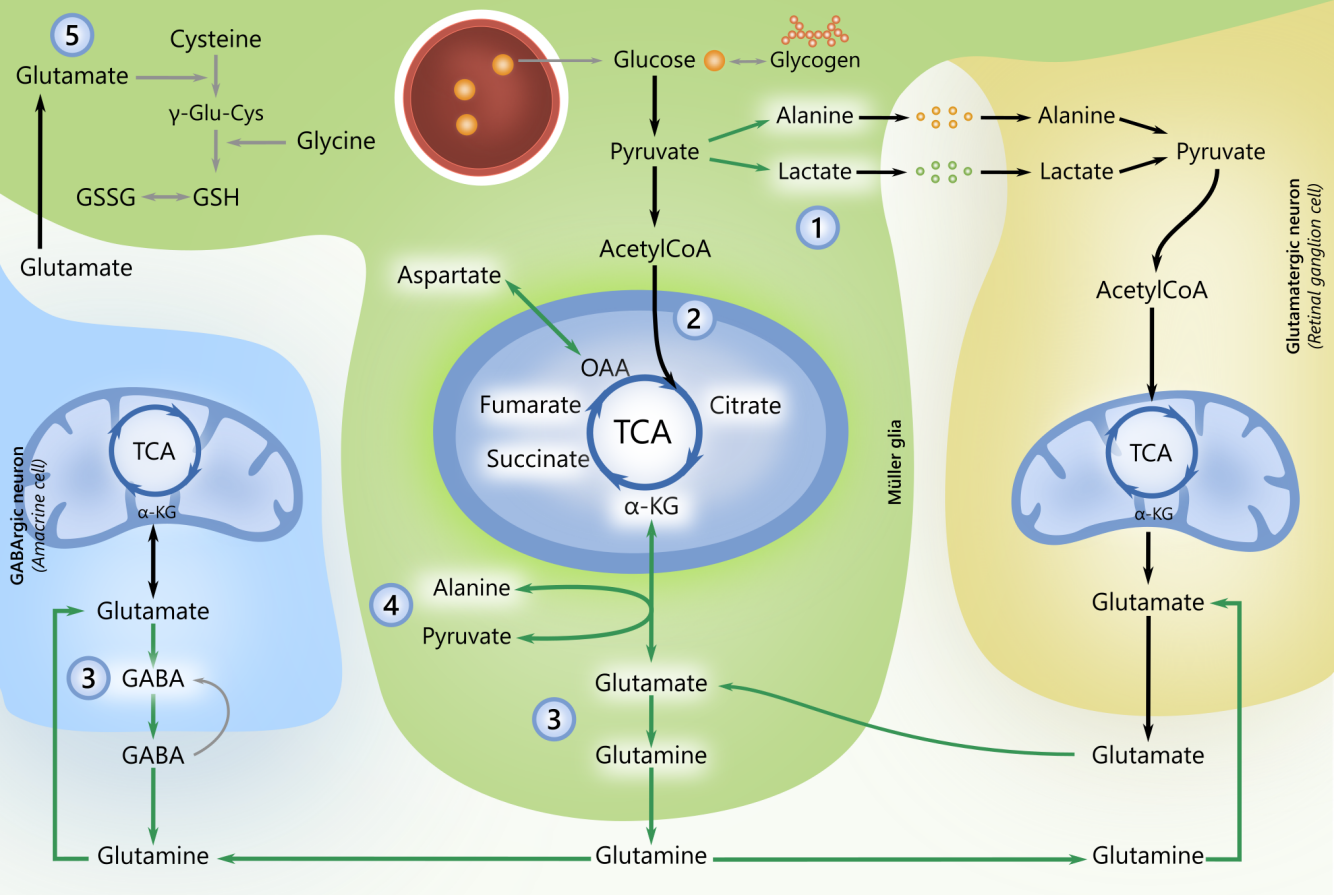
**Schematic summary: Metabolic alterations induced by SEM and the hypothetical mechanisms by which it promotes neuroprotection.** Based on gas chromatography-mass spectrometry (GC-MS) analyses on retinal explants, systemic semaglutide (SEM) treatment increased relative ^13^C enrichment of several key metabolites in glycolysis, the TCA cycle and its associated amino acids. **(1)** Increased glycolysis-associated carbon flux: Through glial-neuronal metabolic interactions, alanine and lactate can serve as auxiliary energy substrates within the retina. SEM increased ^13^C labeling of alanine and lactate, consistent with enhanced incorporation of glucose-derived carbon into glycolysis-associated metabolites. **(2)** Increased TCA cycle-associated carbon flux: SEM treatment increased ^13^C labeling of TCA cycle intermediates (e.g., citrate, α-ketoglutarate (α-KG), succinate, and fumarate) and the associated amino acid aspartate, consistent with increased carbon flux through TCA-linked pathways. **(3)** Improved glutamate-glutamine-GABA cycling: Increased ^13^C enrichment of glutamate, glutamine, and GABA indicates altered routing of glucose-derived carbon toward neurotransmitter-linked pathways, which may support synaptic and metabolic resilience rather than energy production alone. **(4)** Selective metabolic adaptation under mitochondrial stress: In rotenone-exposed retinas, SEM preserved ^13^C labeling of α-ketoglutarate and modestly affected labeling of associated amino acids, including alanine, indicating maintenance of selected mitochondrial- and amino acid-linked carbon flux under complex I inhibition.**(5)** Antioxidant defense systems in the retina: Along with glycine and cysteine, glutamate is used to produce the tripeptide glutathione (GSH). GSH plays a crucial role in the retina's antioxidant defense system by neutralizing reactive oxygen species and maintaining cellular redox balance, by conversion to its oxidized form, glutathione disulfide (GSSG). Despite increasing ^13^C labeling of glutamate, SEM did not reverse ROT induced decrease in the GSH/GSSG ratio. *Green arrows*: elevated activity. GC-MS, gas chromatography-mass spectrometry; SEM, semaglutide; TCA, tricarboxylic acid cycle; α-KG, α-Ketoglutarate; GABA, γ-aminobutyric acid; GSH, reduced glutathione; GSSG, oxidized glutathione (glutathione disulfide); ROT, rotenone.

SEM, a GLP-1RA, ameliorated ROT-induced RGC degeneration following one week of systemic pretreatment. Based on functional metabolic mapping, this neuroprotective effect of SEM appears to be partially attributable to enhanced retinal metabolic flexibility. SEM increased ^13^C labeling of metabolites associated with both glycolysis, the TCA cycle and associated amino acids, indicating greater utilization of alternative energy substrates and preserved mitochondrial carbon flux. Together, these metabolic adaptations suggest that SEM may support RGC survival by increasing availability of alternative energy substrates and facilitating glutamine-glutamate-GABA trafficking (Fig. 7), thereby improving neurotransmitter cycling under mitochondrial stress.
